# The cytoplasmic N-terminal tail of Zika virus NS4A protein forms oligomers in the absence of detergent or lipids

**DOI:** 10.1038/s41598-023-34621-x

**Published:** 2023-05-05

**Authors:** Wahyu Surya, Yiting Liu, Jaume Torres

**Affiliations:** School of Biological Sciences, Nanyang Technological University, 60 Nanyang Drive, Singapore, 637551 Singapore

**Keywords:** Molecular biophysics, Viral proteins

## Abstract

The non-structural (NS) NS4A protein in flaviviruses has three predicted transmembrane domains, is critical for virulence and participates in membrane morphogenesis. In Dengue virus (DENV), both hydrophylic N-terminal tail and its first transmembrane domain participate in the formation of oligomers which are important for pathogenicity. However, the relative importance of the N-terminal domain in oligomerization has been under debate. In particular, since in the absence of detergent or lipids, this domain (residues 1–48) in both DENV and Zika virus (ZIKV) NS4A, was found to be disordered. Recently, however, we reported preliminary data that showed that peptide ZIKV NS4A 4–58 adopts a defined secondary structure in aqueous solution and forms oligomers, signaling its importance for full length NS4A oligomerization. Herein we have performed detailed analytical ultracentrifugation experiments to further characterize the oligomerization of this peptide and also a shorter variant (residues 4–44). In both cases, sedimentation velocity produced a single species with concentration-dependent sedimentation coefficient, consistent with a fast equilibrium between at least two species. Combining sedimentation velocity and equilibrium experiments, data is best fitted to a monomer–dimer–trimer equilibrium. Possible models of NS4A oligomers obtained with AlphaFold-2 predict the stabilizing role for residues in this N-terminal domain, such as Arg20, Asn27, Ala44 and Glu50, all at highly conserved positions in flavivirus NS4A proteins. Our results are thus consistent with N-terminal domain interactions acting as one of the driving forces for NS4A homo-oligomerization.

## Introduction

The *Flavivirus* genus is a group of positive-sense single-stranded RNA viruses that belongs to the family *Flaviviridae*. This genus includes pathogens such as dengue virus (DENV), yellow fever virus (YFV), West Nile virus (WNV) or Zika virus (ZIKV). While DENV infects almost half a billion people every year^1^, other flaviviruses like ZIKV and WNV are also causing significant health concerns^2,3^. ZIKV was first isolated almost 70 years ago^4^ and after its transmission to the Americas, almost hundred countries have experienced outbreaks^5,6^. The majority of ZIKV-infected people are asymptomatic, whereas others suffer influenza-like syndrome and also neurological disorders^7–11^. There are no vaccines nor antiviral agents available to treat ZIKV infections^12,13^ or indeed other flaviviral diseases, and treatment with analgesics and antipyretics is directed to the relief of symptoms^14^. Therefore, the development of vaccines and antivirals against flaviviruses like ZIKV is a current relevant challenge^15^.

After flavivirus infection, a single polyprotein is formed at the endoplasmic reticulum (ER) membrane^16^ which is enzymatically cleaved into structural and non-structural (NS) proteins NS1, NS2A, NS2B, NS3, NS4A, NS4B and NS5. These proteins are crucial in replication, as they form the replication complex (RC) where RNA is synthesized, and ER membrane invaginations, leading to formation of replication organelles (RO).

NS4A is a membrane protein that participates in the scaffold formation of the RC, and is also involved in both RNA replication and the formation of ROs^17–20^, possibly through the formation of a wedge that inserts into the membrane^24^. The interaction of uncleaved precursor NS4A-2K-4B with NS1 is required for viral replication^17,21^. NS4A colocalizes with other proteins of the RC, specifically NS3 and NS4B and viral RNA. Together with NS4B, it is involved in dissociating the RNA from the NS3 helicase domain^22^. The multiple roles of flavivirus NS4A in the infected cell have been summarized recently^23^.

In DENV, NS4A has an extramembrane N-terminal domain encompassing approximately 50 residues, followed by three predicted transmembrane (pTM1-3) segments. Biochemical evidence shows that only pTM1 and pTM3 span the membrane whereas pTM2 is embedded but does not cross the bilayer^20^. This topology is consistent with a faster hydrogen/deuterium exchange observed for pTM2 in solution NMR experiments^24^. A fourth pTM, named fragment 2K, is cleaved in the NS4A-2K-NS4B polypeptide, releasing mature NS4A^20^. Cleavage of this fragment has been found to trigger membrane rearrangements^19,20^. NS4A was sufficient to induce membrane alterations that resembled the typical highly curved RC membranes, possibly mediated by oligomerization^20^. However, although this would be analogous to what has been described for HCV NS4B^25^ and other membrane curvature-inducing proteins^26^, the N-terminal domain of Zika NS4A did not induce liposome aggregation, in contrast to that of HCV NS4B^27^.

This membrane remodeling activity has been linked to NS4A oligomerization. Indeed, flavivirus NS4A has been shown to form homo-oligomers on the basis of multiple bands observed in SDS gels and pull down assays^28–32^, and also hetero-oligomers with other viral proteins, such as NS4B^31^. The more hydrophilic N-terminal domain was first suggested to be important for homo-oligomerization^30^. This hydrophilic domain has been mutated extensively and is important for replication and viral production^18,33^. Other studies based on pull down assays have shown that pTM1 (residues ~ 50–76) may be the main contributor^32^ to oligomerization. However, the latter study used bulky green fluorescent protein fused to various NS4A fragments, which may interfered in the interactions observed. The interaction with NS4B also appears to involve at least part of the N-terminal tail^30,32^ together with pTM1, suggesting that these homo- and hetero-meric interactions cannot take place simultaneously. NS4A oligomerization may be triggered by the cleavage of peptide 2K, which in turn may induce membrane remodeling. Thus, disruption of these inter-molecular interactions constitutes a potential strategy for antiviral drugs^22,34,35^.

The similarity between NS4A in DENV and ZIKV predicts that they have similar characteristics. Indeed, they have exactly the same length (127 residues), predicted topology and TM regions^36^. In DENV, several oligomeric forms have been observed in gels; monomers, dimers and higher oligomers, possibly tetramers^24,31,32^.

The critical role of NS4A N-terminal tail has led to the exploration of its structure in both DENV and ZIKV. This tail was reported to be unstructured in solution, and formed α-helices upon interaction with detergent micelles or membranes^30,37–39^. Despite these reports, we showed recently that a peptide from ZIKV NS4A that spans residues 4–58, which along with full length NS4A was found to express efficiently in *E. coli* using a simple His-tag, could form oligomers even in aqueous solution^27^. This peptide includes most of the N-terminal tail and a third of its first TM domain, and formed substantial α-helical structure in water as measured by circular dichroism (CD). While the oligomeric size was estimated to be trimeric from gel filtration and static light scattering, these techniques have low resolution, and this behavior was not characterized further. In the present paper, we have used analytical ultracentrifugation (AUC) in both sedimentation equilibrium (SE) and sedimentation velocity (SV) modes to confirm and further define this behavior. AUC measures protein–protein interactions in solution and does not require calibration or interaction with matrices. The relevance of the hydrophilic N-terminal domain for NS4A oligomerization is discussed.

## Methods

### ZIKV NS4A N-terminal peptide expression and synthesis

**Figure 1:**
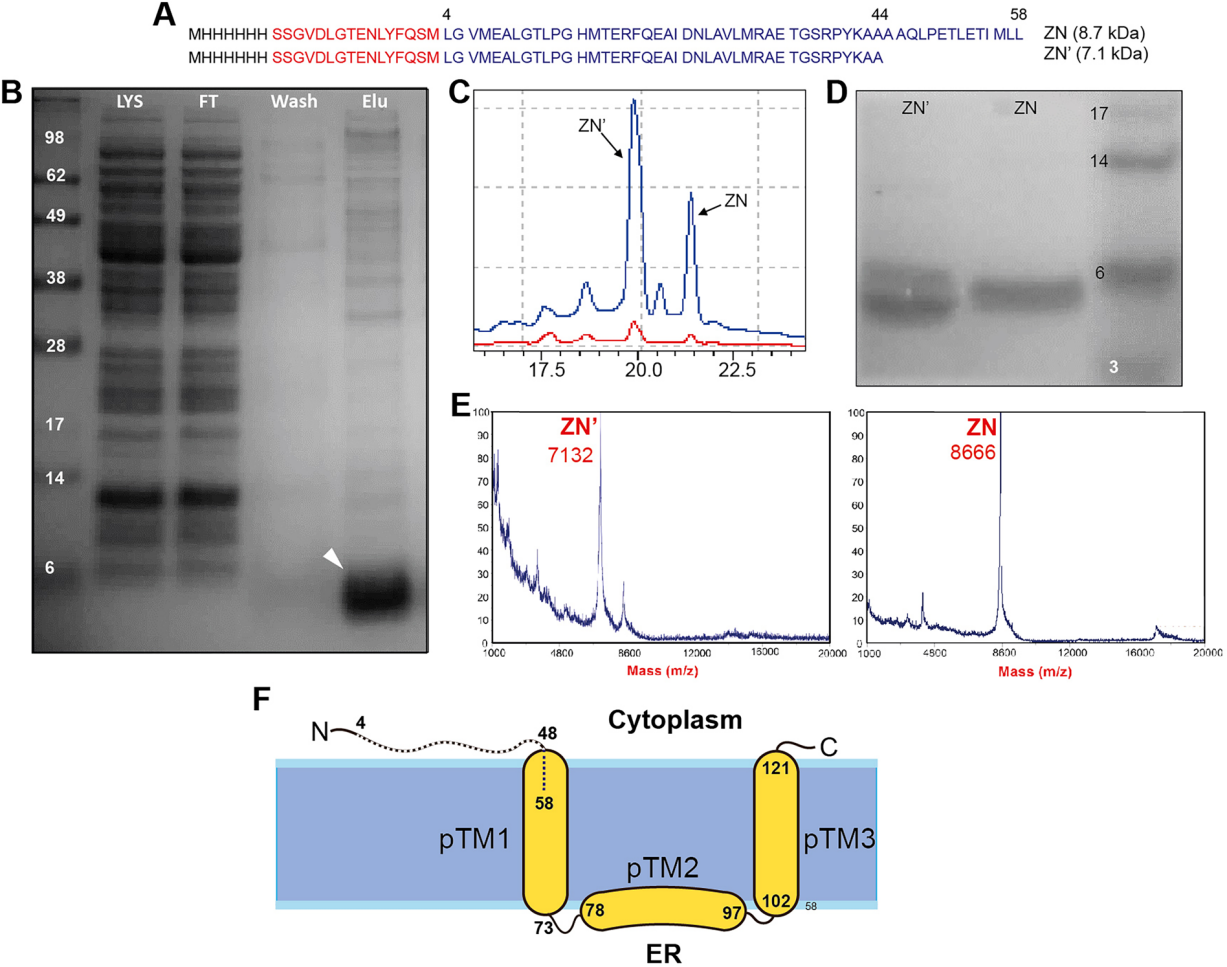
Purification of ZIKV NS4A ZN peptide. (**A**) Sequence of purifed ZN peptide, with His tag (black), linker (red) and residues 4–58 of NS4A (blue). The sequence of truncated ZN′ is also shown; (**B**) NuPAGE of the Ni–NTA fractions, where LYS, FT, Wash, and Elu are lysate, flow-through, wash and elution from the IMAC column, respectively. The white arrowhead points to the ZN peptide band; (**C**) HPLC chromatogram of ZN peptide; (**D**) NuPAGE gel image of the HPLC fractions in (**C**); (**E**) MALDI-MS spectra of the fractions in (**C**) with molecular weights indicated; (**F**) schematic representation of ZIKV NS4A, where the topology and helical domains are assumed to be the same as in NS4A DENV^20,24^. The N-terminal peptide ZN (4–58) used in the present work is indicated as a dotted line. Gels in panels (**B**) and (**D**) were cropped from complete gels shown in Supplementary Fig. S1. The illustration in (**F**) has been made in Adobe Illustrator.

The N-terminal peptide construct (residues 4–58) of Zika NS4A protein (strain MR-766, accession YP_002790881.1), hereafter named ZN, was expressed and purified with an N-terminal His-tag (Fig. 1A), as described^27^. Immobilized metal-affinity chromatography (IMAC)-eluted fractions were precipitated with trichloroacetic acid (TCA) and washed with acetone twice. The final pellet was dissolved in 50% acetonitrile (ACN) and lyophilized. The peptides were further purified using ACN gradient on a C4 reversed-phase HPLC column (Phenomenex). Fractions were identified by MALDI-TOF mass spectrometry, pooled, and lyophilized in 50% ACN with 1 mM HCl to remove residual TFA. Samples were stored at − 20 °C until further use. Peptide (4–44) was synthesized by Genscript (Singapore) and purified by HPLC as described above.

### Gel electrophoresis and protein labeling

Measurement of protein concentration and SDS-NuPAGE were performed as previously described^27^. Fluorescently tagged ZN peptides were labeled using Alexa Fluor 555 NHS Ester (ThermoFisher Scientific) according to the manufacturer’s protocol.

### Analytical ultracentrifugation (AUC)

AUC data were collected on a Beckman ProteomeLab XL-I Analytical Ultracentrifuge with a 4-place An-60 Ti or 8-place An-50 Ti analytical rotors. Samples for sedimentation velocity (SV) experiments were prepared in 50 mM Tris–HCl (pH 7.4) in the approximate range 3–300 µM. Samples were loaded into AUC cells fitted with 2-sector Epon centerpiece and quartz windows. The cells were centrifuged at 50,000 rpm at 20 °C and absorbance scans at 230 or 280 nm, as indicated, were collected every 5 min. The data were analyzed using *c*(*s*) and *c*(*s,ff0*) size distribution models in SEDFIT^40^ and hybrid local continuous global discrete model in SEDPHAT^41^.

For sedimentation equilibrium (SE) experiments, the peptide was dissolved at approximately 10, 20, and 40 µM, as indicated, in 50 mM Tris–HCl pH 7.3, 150 mM NaCl. Samples were loaded into AUC cells fitted with 6-sector epon centerpiece and quartz windows. Multi-speed equilibrium profiles at 280 nm was collected after centrifugation at 23,000, 28,000, 34,500, and 42,000 rpm for about 24 h at each speed. Approach to equilibrium was tested using the Match utility in Heteroanalysis^42^. SE data were fitted to self-equilibrium models in SEDPHAT^43^. Buffer density and viscosity, and protein partial specific volume were calculated using SEDNTERP^44^. Data was graphically presented with GUSSI^45^.

### Modelling of the ZN peptide and full length NS4A with AlphaFold-2

Models of peptide ZN and full length NS4A were obtained with ColabFold (AlphaFold2 using MMseqs2)^46^ using the sequence of ZIKV NS4A 1–58 (59 residues), from monomer to homo-pentamers, or 1–127 (full length NS4A). Parameters used were *use_amber* = True, *template_mode* = None, *msa_mode* = MMSeqs2(Uniref + Environmental), *pair_mode* = unpaired + paired, *model_type* = auto, *num_recycles* = 24. For each prediction, best models (rank 1) were selected, according to average pLDDT, and complexes were sorted by pTMscore. Hydrodynamic parameters for each ZN model were calculated using HullRad^47^ implemented in SViMULATE software^48^.

## Results and discussion

### Purification of ZN peptide

The ZN peptide (Fig. 1A) was expressed and purified first using an IMAC Ni-affinity column, where it appeared as a single band on SDS-NuPAGE gels, with some impurities (Fig. 1B). The band showed anomalous migration, slightly faster than its theoretical molecular weight (8.7 kDa), as expected from peptides that interact with lipids^27^. In our previous report^27^ this peptide was purified further by size exclusion chromatography (SEC) using Superdex 200 increase 10/300 GL column. Elution at 15.4 mL was consistent with the formation of an oligomer, possibly a trimer, similar to the one observed for the full length protein. Here, we used HPLC instead to obtain a purer ZN peptide. Two HPLC fractions contained peptide (at 19.7 and 21.5 min) (Fig. 1C). The latter fraction consisted of pure ZN peptide, as shown by SDS-NuPAGE (Fig. 1D) and mass spectrometry (Fig. 1E). The peptide appearing first, at 19.7 min, was ZN truncated at the C-terminus (18 residues missing) and is termed here ZN′ (Fig. 1A, 7.1 kDa). The final yield of ZN and ZN′ peptides was 3 and 9 mg/L of TB culture, respectively.

### Sedimentation velocity of ZN and ZN′ peptides

**Figure 2:**
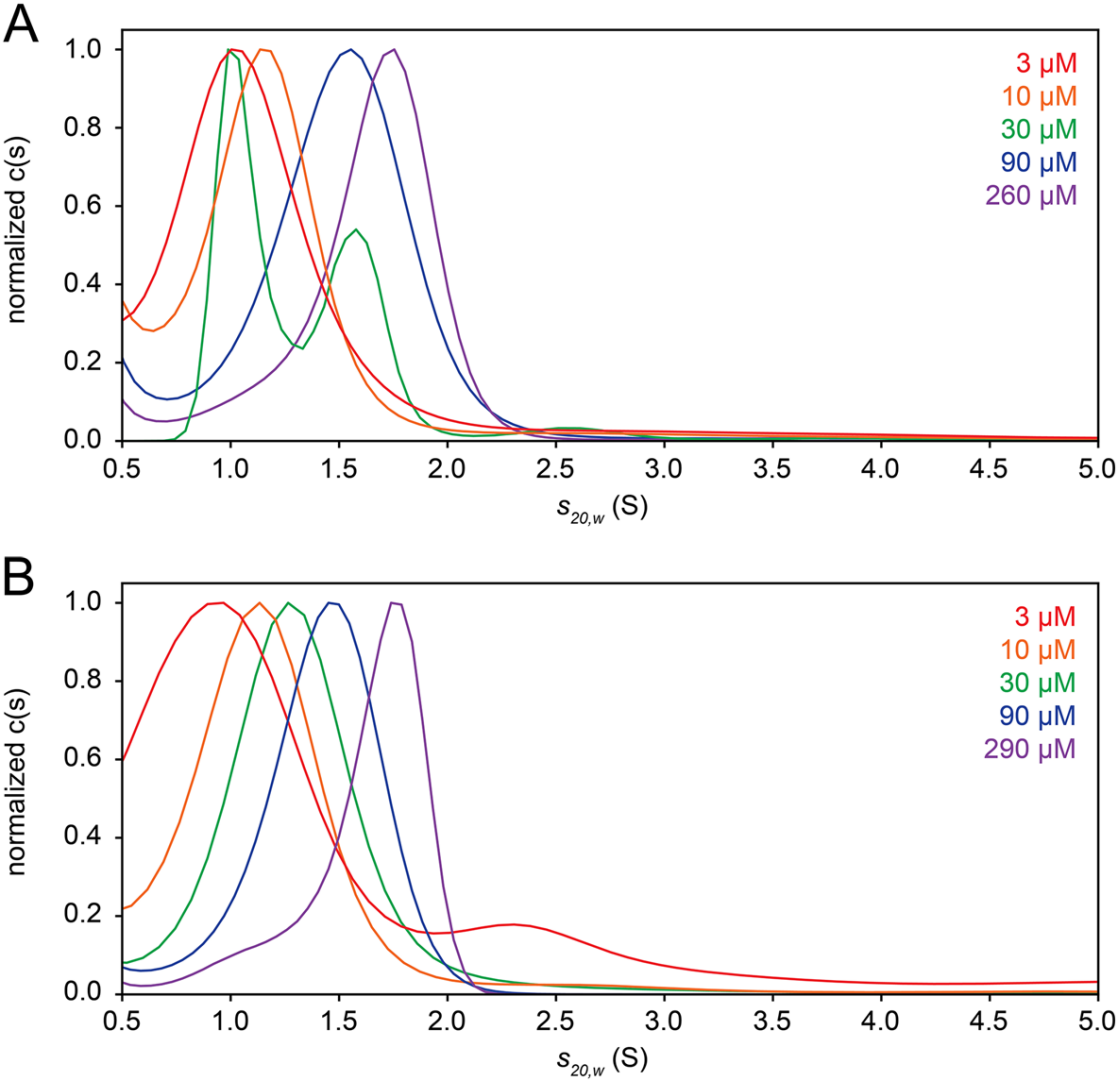
Size distribution *c*(*s*) of ZN and ZN′ peptides. (**A**,**B**) Normalized *c*(*s*) distribution plots corresponding to peptide ZN (**A**) and ZN′ (**B**), colour-coded to the respective loading concentrations indicated on the top-right corner.

We suggested previously that the ZN construct (Fig. 1A) forms homotrimers in solution^27^, based on static light scattering results and using concentrations which ranged from 0.05 to 1.2 mg/mL (approximately 6–160 µM). However, dynamic and static light scattering (DLS ad SLS, respectively) do not provide sufficient resolution to confirm this claim. To test this, herein we first performed sedimentation velocity (SV) experiments on ZN peptide at a broader concentration range (3 to ~ 300 µM) (see raw data in Supplementary Fig. S2). In the corresponding normalized c(s) plots (Fig. 2A), a single band was observed which clearly shifted with increasing concentration. From 3 to 260 µM, the sedimentation coefficient, S, increased from approximately 1.0 to 1.75. This concentration-dependent S value is encountered in conditions of rapid exchange, where the S value observed at each concentration is a weighted average of smaller and larger individual components^49^. Using Sedfit, from the S value and frictional ratio, the estimated molecular weight at the lowest concentration is 14.3 kDa, i.e., intermediate between theoretical monomer (8.7 kDa) and dimer (17.3 kDa), and at the highest concentration is ~ 17.5 kDa, consistent with a dimer. These SV experiments and analyses were also performed on the truncated peptide ZN’ (7.1 kDa) with similar results (Fig. 2B): a single band shifted to higher S values with increasing concentration, from predicted 14.3 kDa (dimer) at 10 µM to 17.5 kDa (larger than a 14.2 kDa dimer) at 290 µM. In both ZN and ZN′, we used a more diluted sample (3 µM) which produced a lower S value, corresponding to a molecular weight between that of monomer and dimer. However, the low signal in these 3 µM samples precluded a more detailed c(s, ff0) analysis (see below).

Outside the concentration range used herein, the bands may still shift further towards the size of a monomer (< 3 µM) or perhaps a trimer (> 300 µM), but low signal and reduced solubility prevented the use of these more extreme concentrations.

In an attempt to probe the behaviour of ZN peptide at concentrations below 3 µM, we labeled the ZN peptide with the fluorophore Alexa Fluor 555. However, unlike the unlabeled peptide, the *c*(*s*) profile did not show any concentration dependency between 6 and 24 µM (Supplementary Fig. S3), sedimenting as a single species at 1.11 S. The expected molecular weight for this species (11.3 kDa) corresponds to the monomeric ZN peptide tagged at two positions, likely the N-terminus and residue Lys42, and suggests that the fluorophore tag interfered with ZN peptide oligomerization. Thus, this approach was abandoned.

### Size-and-shape distribution c(s, ffo) analysis of ZN and ZN′ peptides

**Figure 3:**
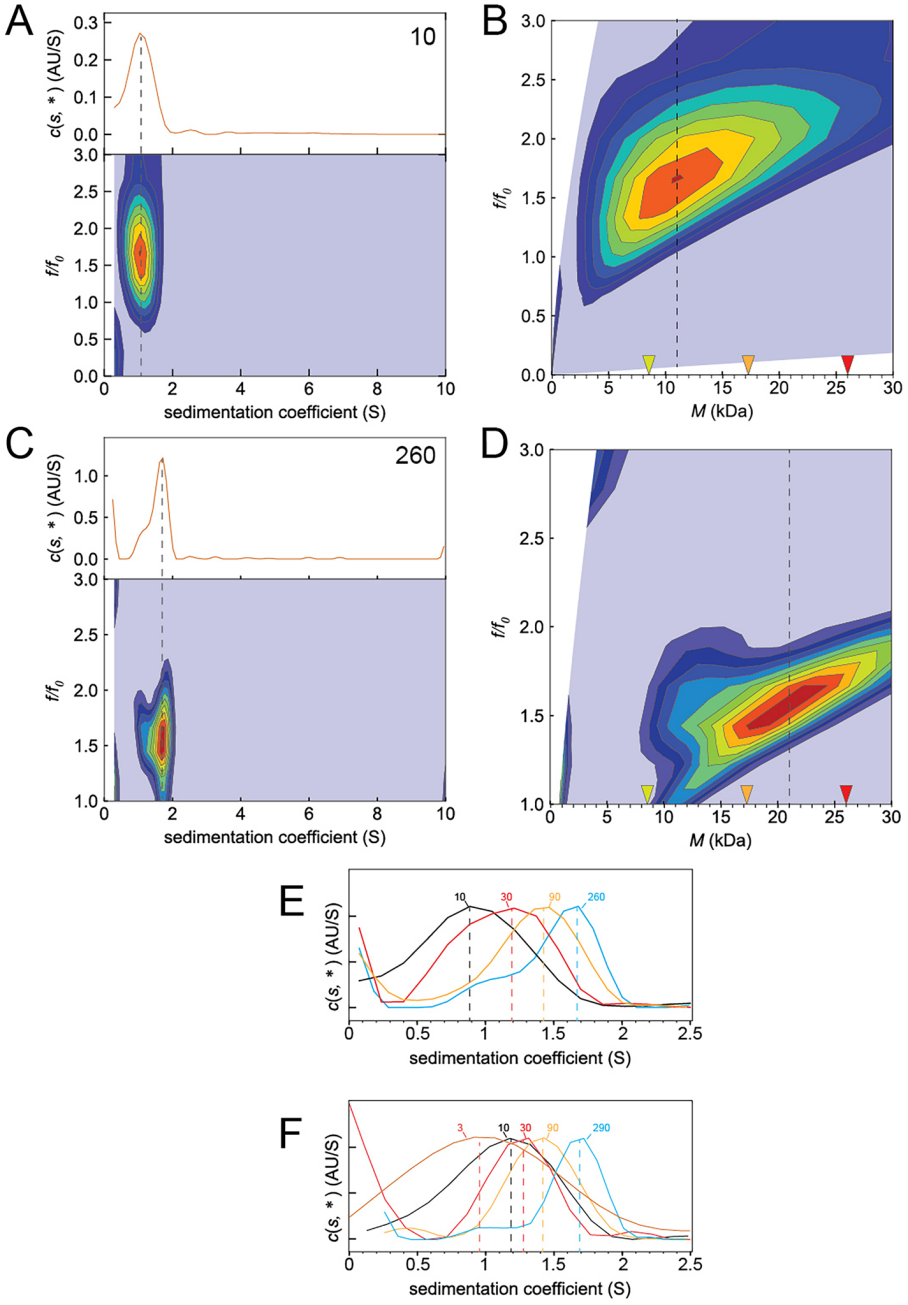
Size-and-shape *c*(*s, ffo*) distributions for ZN and ZN′ peptides. (**A**,**B**) Example of plot of S versus f/fo (**A**) and molecular weight (M) versus f/fo (**B**) at 10 µM ZN peptide. The *c*(*s*, *) projection is shown at the top of panel (**A**). In (**B**), theoretical molecular weights of monomer, dimer, and trimer are indicated with inverted triangles yellow, orange, and red, respectively; (**C**,**D**) same as (**A**,**B**), for the highest ZN concentration tested, 260 µM; (**E**) *c*(*s*, *) plots overlay for all ZN peptide concentrations tested; (**F**) *c*(*s*, *) plots for all ZN′ peptide concentrations tested.

The average molecular weight obtained in the previous section was predicted by Sedfit based on a uniform f/fo ratio (shape parameter) for all species present in the sample. However, a more accurate analysis is performed by considering that the f/fo ratio is a species-dependent parameter. Thus, the average molecular weight was obtained again after a more complex size-and-shape *c*(*s, ffo*) analysis, where the f/fo ratio was included as a variable in a second dimension, and the species population was in a third dimension. The average molecular weight in the ZN and ZN′ samples was derived from these more accurate f/fo values, using a projection, *c*(*M*), where molecular weight (M) is plotted against f/fo (see Fig. 3 and Supplementary Figs. S4–5). For ZN 10 µM, the c(s, ffo) plot, the projection c(s, *) (Fig. 3A) and its corresponding c(M) plot (Fig. 3B) indicate an average between monomer and dimer. For the highest concentration tested, 260 µM (Fig. 3C,D), the average is between dimer and trimer. An overlay of the *c*(*s*, *) plots for each ZN concentration (Fig. 3E) shows the shift in S value with concentration. Similar results were obtained with the truncated peptide ZN′ (Supplementary Fig. S4) and an overlay of *c*(*s*, *) plots for each ZN′ concentration is shown in Fig. 3F. Thus, for both ZN and ZN′, the average molecular weight at the highest concentration tested (~ 270) µM was in between that of a dimer and a trimer.

Lastly, since similar results were obtained with peptides ZN and ZN′, we synthesized and purified a peptide corresponding to residues 4–44, i.e., without His-tag or linker sequence (Supplementary File S6). This peptide was also found to form oligomers of increasing size depending on concentration, although with lower affinity (Supplementary Fig. S7).

### Non-interacting species

**Figure 4:**
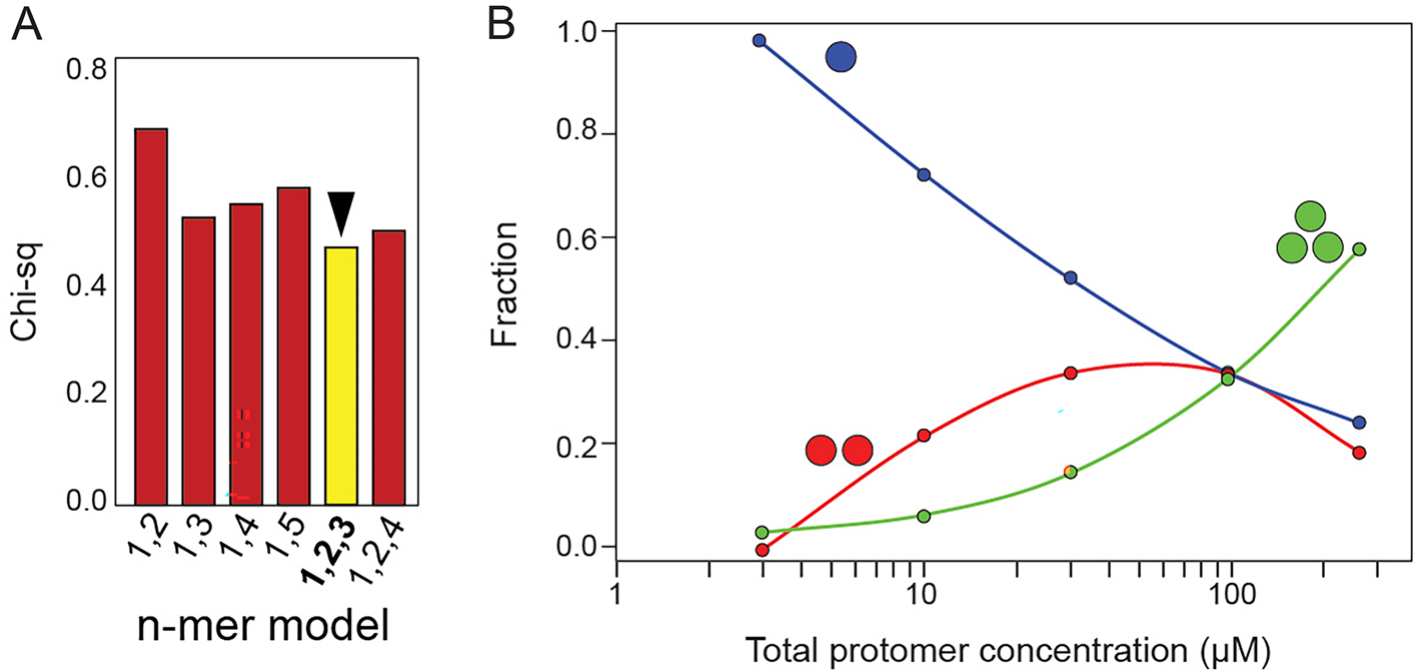
Analysis of ZN peptide SV data using a non-interacting model. SV profile of ZN peptide at 3, 10, 30, 90, and 260 µM were fitted to a hybrid local continuous global discrete model, in which the species involved are non-interacting. (**A**) Comparison of Chi-squared values for various discrete species compositions. The best fit model (yellow) contains monomers, dimers and trimers; (**B**) species distribution for the non-interacting best fit model, yellow in (**B**), with monomers (blue), dimers (red) and trimers (green).

The SV data was analysed further using a non-interacting species model in SEDPHAT. Fitting to a hybrid local continuous global discrete model revealed that the mixture likely contains the discrete species: monomer, dimers and trimers (Fig. 4A). In the range 3–300 µM, the proportion of monomer decreases gradually, the dimer first increases and later decreases, whereas the trimer keeps increasing (Fig. 4B). Thus, the underlying presence of these species is consistent with the observed concentration-dependent single band in *c*(*s*) and *c*(*s, ff0*) profiles (Figs. 2, 3). We note that, since this is a non-interacting model, the relative populations in Fig. 4B do not represent those present in the sample. Rather, this model is a rough indication of the *types* of oligomers present.

### Sedimentation equilibrium (SE) of ZN peptide

**Figure 5:**
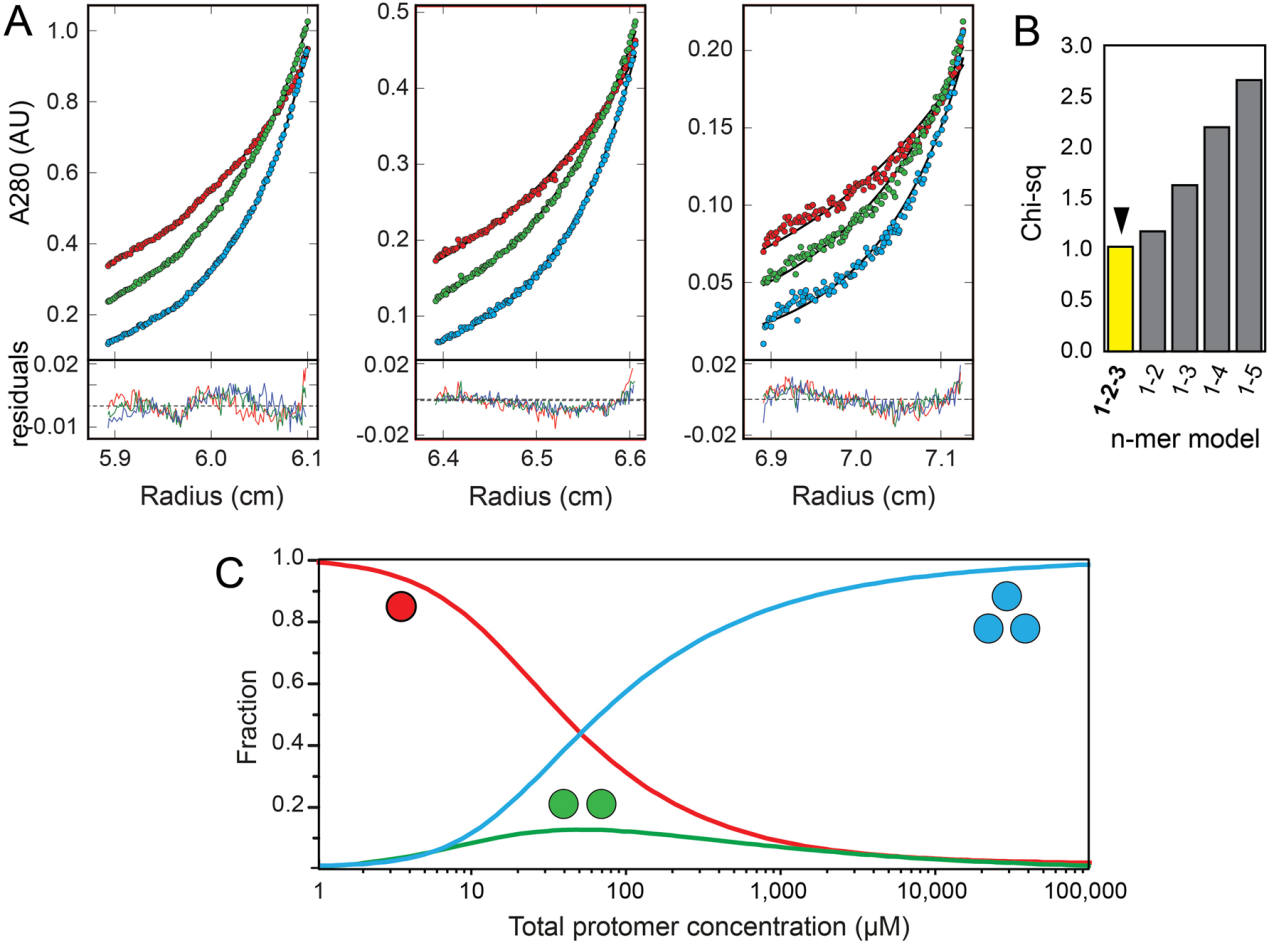
Sedimentation equilibrium of ZN peptide. (**A**) Sedimentation profile of ZN peptide (circles) loaded at initial concentrations (from left to right) 40, 20, and 10 µM. The solid line below the circles corresponds to the best fit 1–2–3 model (see **B**). Fitting residuals are shown in the lower panel; (**B**) Chi-squared values of the oligomerization models tested, shown in the x-axis; (**C**) species population plot corresponding to the best-fit 1–2–3 model using the SE data (**A**).

The oligomerization behaviour of the ZN peptide was examined further using SE data (Fig. 5A). The best fit model to the data was a monomer–dimer-trimer equilibrium (1–2–3) (Fig. 5B), consistent with the SV data shown above. Indeed, removing the dimeric form invariably led to a poorer fit, but in a species population plot this dimeric form was very small (not shown). However, when the highest speed data was removed, the species population plot (Fig. 5C) showed the predicted concentration fractions of these oligomers as a function of total peptide concentration, with the dimer as intermediate species, suggesting that the simple model used cannot fully explain the complexity of this system. We further note that the actual dimer population depends on the data range chosen and initial equilibrium guess values.

### Predicted structures of the N-terminal tail of ZIKV NS4A

**Figure 6:**
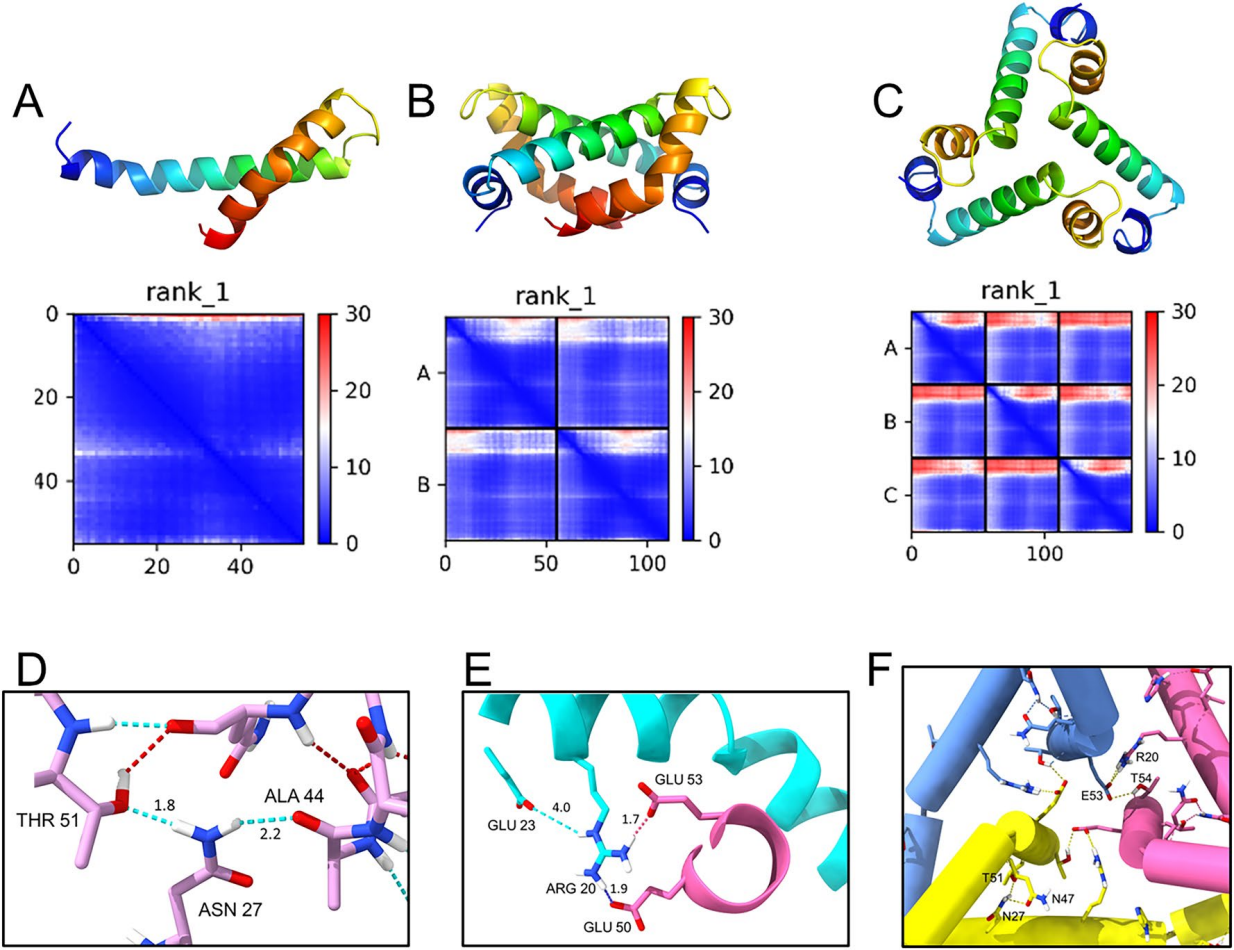
ZN structure prediction with Alphafold-2. (**A**–**C**) Predicted AF2 structures for peptide (4–58, i.e., without His tag and linker), from N-terminus (dark blue) to C-terminus (orange) for monomer (**A**), dimer (**B**) and trimer (**C**). Below, the Predicted Alignment Error (PAE) plots show the level of confidence in long range interactions (more confidence = blue, less confidence = red). Most of the residues in the AF2 had high pLLDT confidence scores (pLDDT > 80); (**D**–**F**) critical interactions predicted by AF2 within the region 1–58 of full length ZIKV NS4A monomer (**D**), dimer (**E**) and trimer (**F**).

We attempted the prediction of the structures of ZN or ZN′ (Fig. 1A) monomers, dimers, and trimers using AlphaFold-2^50,51^, but none of the models were reliable (not shown). However, good quality models for monomers, dimers and even trimers were obtained after His-tag and linker were removed (Fig. 6A–C). The quality of these three models according to Predicted Alignment Error (PAE) plots is high (dark blue), but rapidly deteriorated for higher oligomers (not shown). The homotrimer shown in Fig. 6C (residues 4–58) was predicted to have S value of 1.90 and f/fo = 1.2. This frictional ratio is significantly lower than the one obtained experimentally with the ZN peptide, consistent with the removal of the His-tag and linker. Indeed, the best trimeric ZN obtained with AF2 (residues 4–58 with His-tag and linker) (not shown) had an f/fo ratio of 1.7 and a predicted S value of 1.86 (calculated in SViMULATE), which are values consistent with the experimental SV results (Figs. 2, 3). However, we note that the accuracy of these models is doubtful since the dimeric model (Fig. 6B) was different when using other flavivirus NS4A sequences such as DENV or WNV (not shown), or when using the full length ZIKV NS4A in AF2 (see Fig. 6D–F). Additionally, trimeric oligomers of peptide 4–58 were not found, or were different, using other flavivirus NS4A sequences of peptide 4–58. This could be caused by the small number of sequences used in the aligment when using short sequences. Therefore, we used the full length ZIKV NS4A sequence in AF2 to produce models of monomers, dimers and trimers where critical potential interactions are shown in Fig. 6D–F, respectively. For the monomer, hydrogen bonds are shown between residues Asn27, Ala44 and Thr51 (Fig. 6D). Asn27 is always a polar residue in all flavivirus NS4A sequences (see Supplementary Fig. S8), either Asn or Thr, whereas Ala44 is fully conserved. For the dimer (Fig. 6E), interactions involve a salt bridge between Arg20 in one monomer and Glu50 and Glu53 in another. Arg20 is always a basic residue (either Lys or Arg), whereas Glu50 is always an acidic residue (Glu or Asp), except in BUSV sequence which is very distant. Glu53 is always Glu or other polar residues in other sequences (Thr, Ser, Gln). The trimer produced a less reliable model (not shown) but interactions again involved Arg20 in one monomer and Glu53 in another.

Overall, it is clear from SV and SE experiments that oligomers of ZN are present in solution, likely up to homotrimers. Additionally, since a disordered peptide cannot form oligomers, these results confirm that the N-terminal domain of NS4A in ZIKV, an likely all other flaviviruses, is folded in aqueous solution, as shown previously using CD^27^. We previously hypothesized that this oligomerizing behavior was due to the inclusion of residues that belong to one third of the first TM domain of NS4A (residues 49–58), a region that includes residues involved in intermonomer interactions (Fig. 6E,F). However, the shorter version ZN′ was also able to form oligomers, even when the N-terminal His-tag was absent.

Although in the literature, the oligomeric size of NS4A has been proposed to be a dimer, this conclusion is only supported by bands in SDS gels, which often do not represent the behaviour in milder detergents or in lipid membranes. Our present results show that the N-terminal tail of Zika NS4A is able to form up to trimers, which is consistent with our previous estimate for full length NS4A based on the elution volume in gel filtration^27^. Although monomer–monomer affinity is rather low and complete trimerization seems to require close to mM concentration, nevertheless, at 50 µM, 50% of the monomers form trimers (Fig. 5C). Thus, the present AUC data supports the role of the N-terminal domain in promoting NS4A oligomerization. Although the final size of the NS4A oligomer is unknown, the present data supports that it should be at least trimeric. The N-terminal cytoplasmic tail of ZIKV NS4A is unlikely to be glycosylated since it is exposed to the cytoplasm, and no glycosylation of NS4A has been reported experimentally^52^. Thus, using a recombinant peptide obtained in *E. coli* like the one used here may form the basis of a useful in vitro drug screening assay.

## Conclusion

We have shown that peptide ZN, encompassing the N-terminal residues 4–58 of ZIKV NS4A protein (54 residues) can be purified in good yield. This peptide, as well as its truncated form (residues 4–44), forms oligomers, up to trimers, in aqueous solution without the need of detergent or lipid membranes. A more detailed analysis of these homomeric interactions requires structural determination and mutation analysis that is out of the scope of the present work.

## Supplementary Information

Supplementary Figures.

## Data Availability

The datasets generated during and/or analysed during the current study are available from the corresponding author on reasonable request.
