# Supplementary material for: The cytoplasmic N-terminal tail of Zika virus NS4A protein forms oligomers in the absence of detergent or lipids
Source: Sci Rep. 2023 May 5;13:7360. doi: 10.1038/s41598-023-34621-x (PMC10163220; doi:10.1038/s41598-023-34621-x)
Supplement: Supplementary file 1 — Supplementary Figures. [file 41598_2023_34621_MOESM1_ESM.docx]

**Supplementary File.**

Wahyu Surya, Yiting Liu and Jaume Torres *


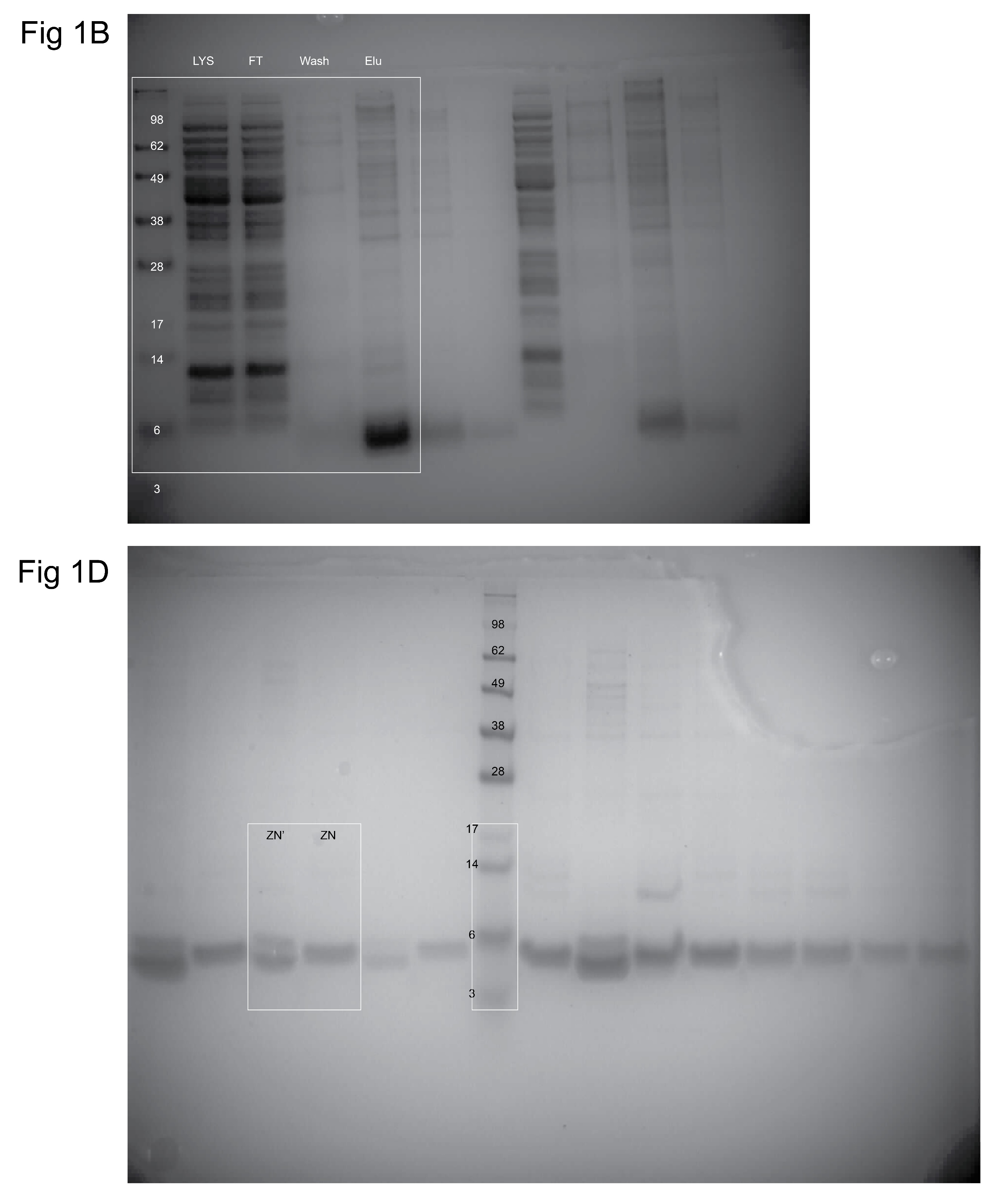


**Figure S1. Complete gels corresponding to Fig. 1B and Fig. 1D in the manuscript.** Areas cropped in the gel are shown inside white rectangles.


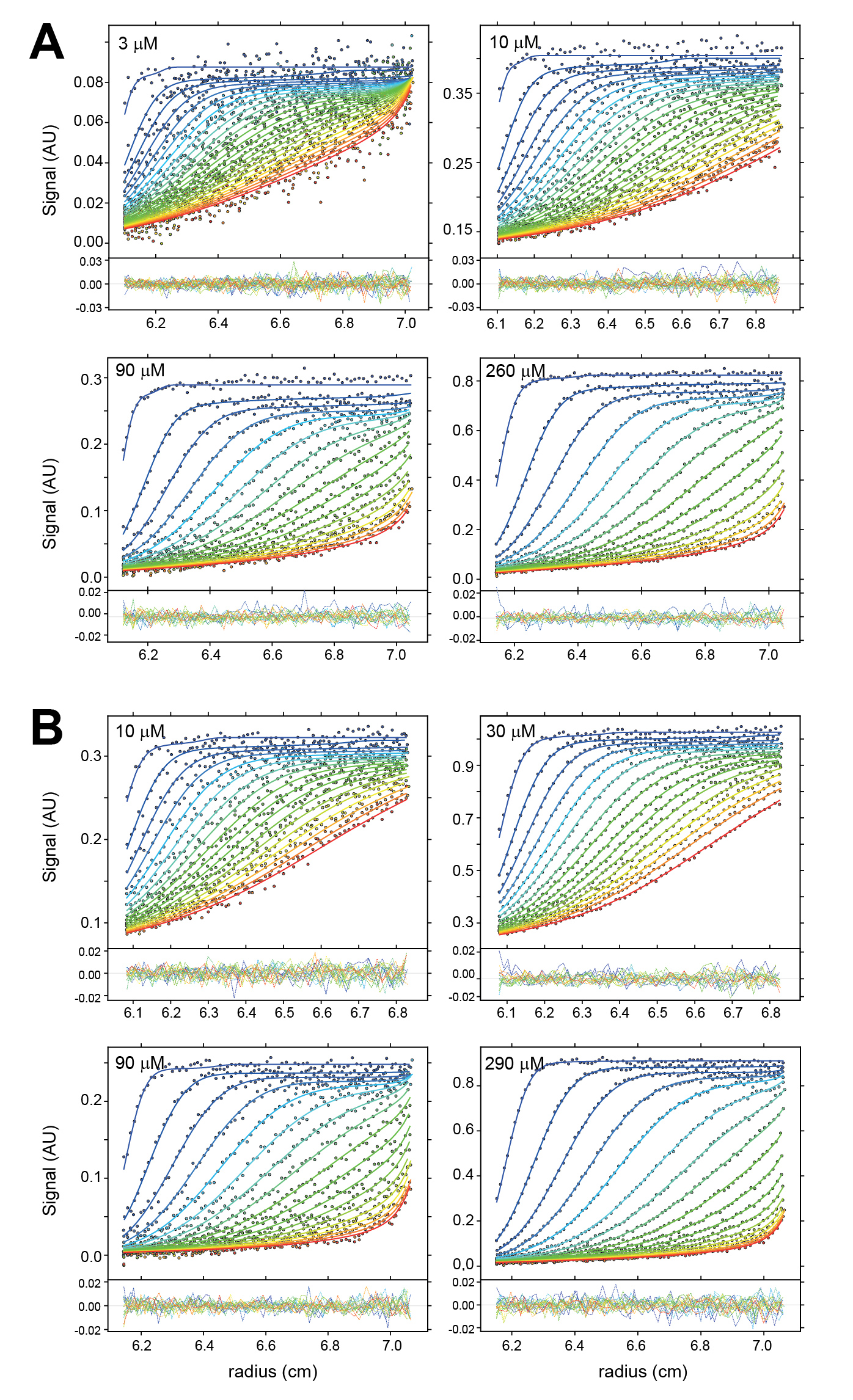


**Figure S2. Examples of sedimentation velocity (SV) profiles of ZN and ZN’ peptides.** (A-B) SV raw data collected at 280 nm corresponding to ZN (A) and ZN’ (B) peptides. Concentrations (µM) are shown in the upper left corner. Data at concentrations lower than 20 µM was obtained at 230 nm.


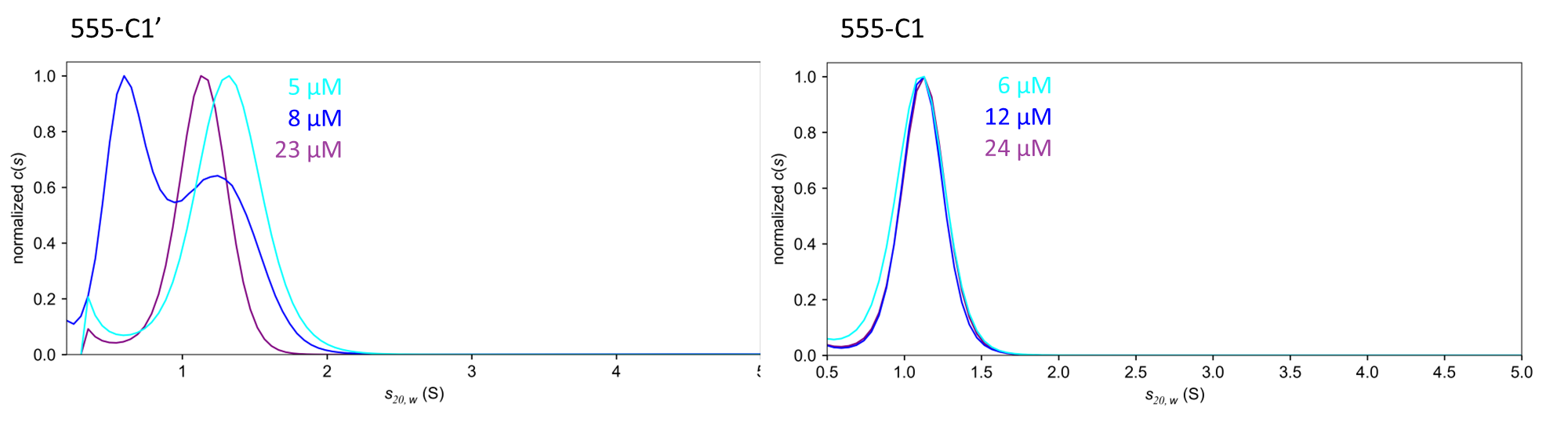


**Figure S3. Size distribution *c(s)* of fluorescently labeled ZN peptide.** c(s) plots corresponding to SV experiments performed in the range 6 and 24 µM for ZN peptide labeled with Alexa Fluor 555.


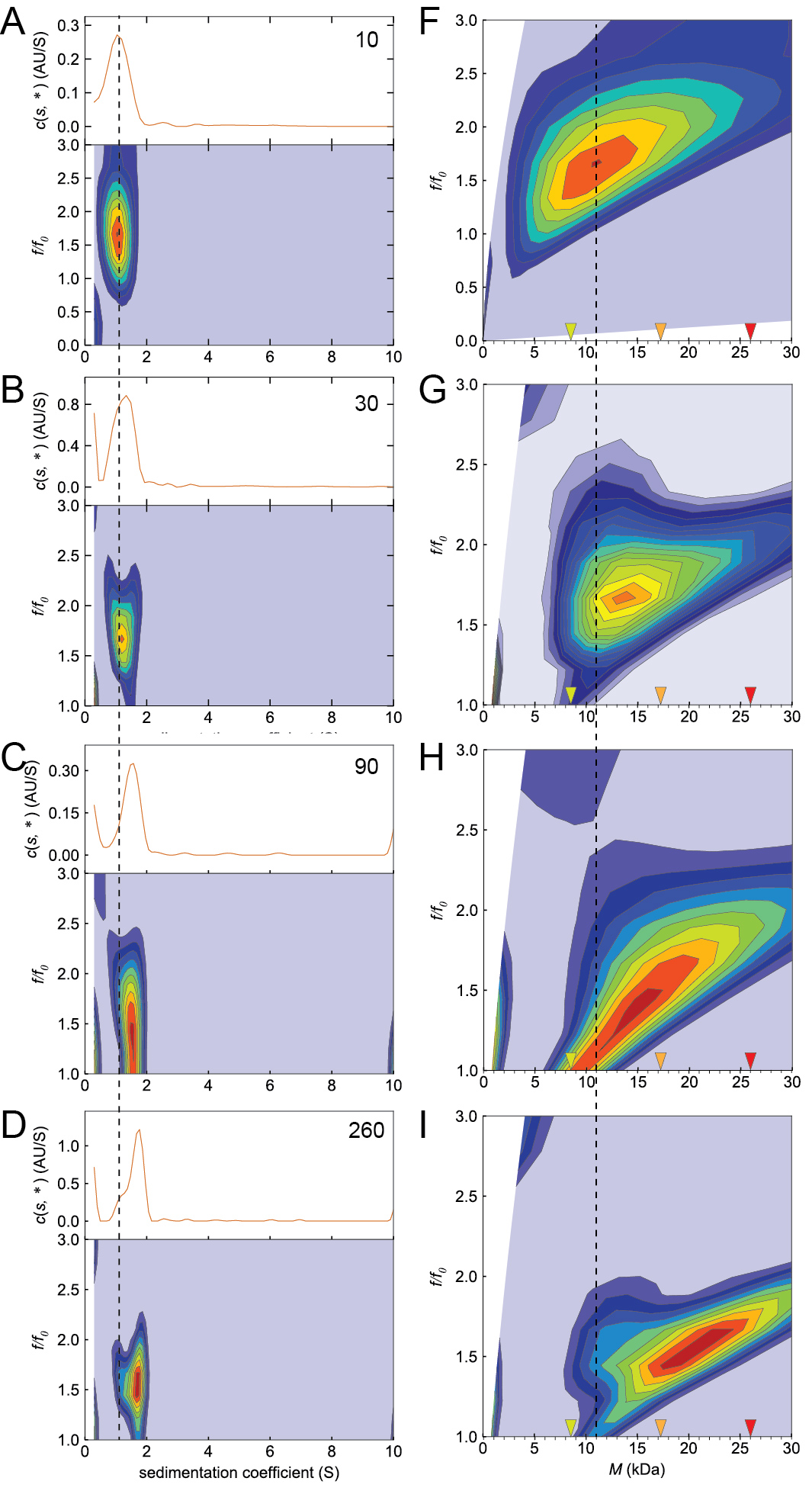


**Figure S4. Size-and-shape *c(s, ffo)* distribution of ZN peptide.** (A-D) Plots of S versus f/fo at the ZN concentrations (µM) shown at the top-right of each panel. The *c(s, *)* projection is shown above each panel; (E) *c(s, *)* plots from panels A-D are overlapped for comparison; (F-I) plots of molecular weight (M) versus f/fo, where the theoretical molecular weights of monomer, dimer, and trimer are indicated with inverted triangles yellow, orange, and red, respectively.


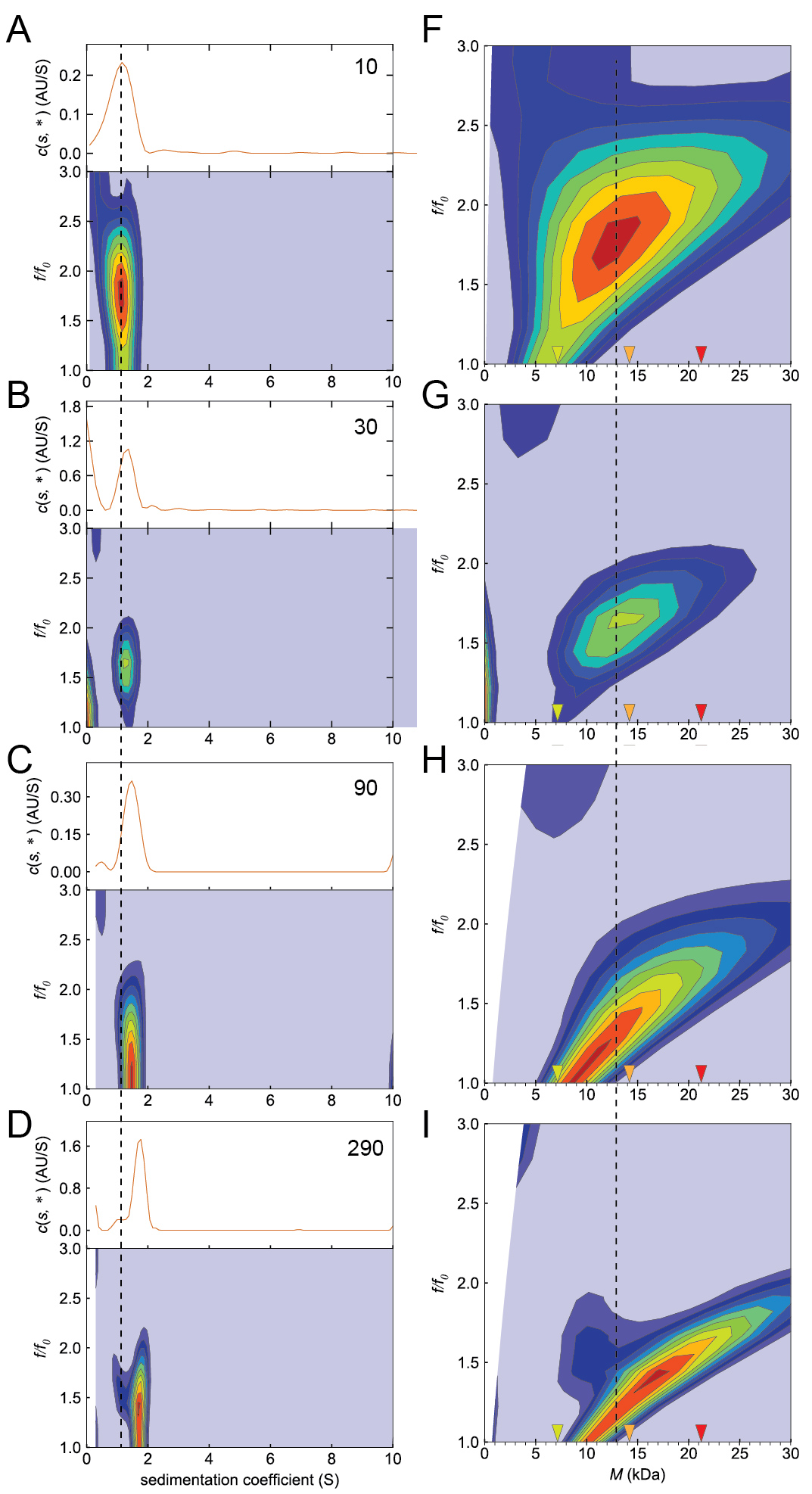


**Figure S5. Size-and-shape *c(s, ffo)* distribution of ZN’ peptide.** Panels are the same as in Fig. 4, for ZN’ peptide. In panel E, the *c(s, *)* plot for 3 µM has been added to show that even at this concentration there was a shift in the S value, but the data from this sample was too noisy for the 2D representation (panels A-D).

**
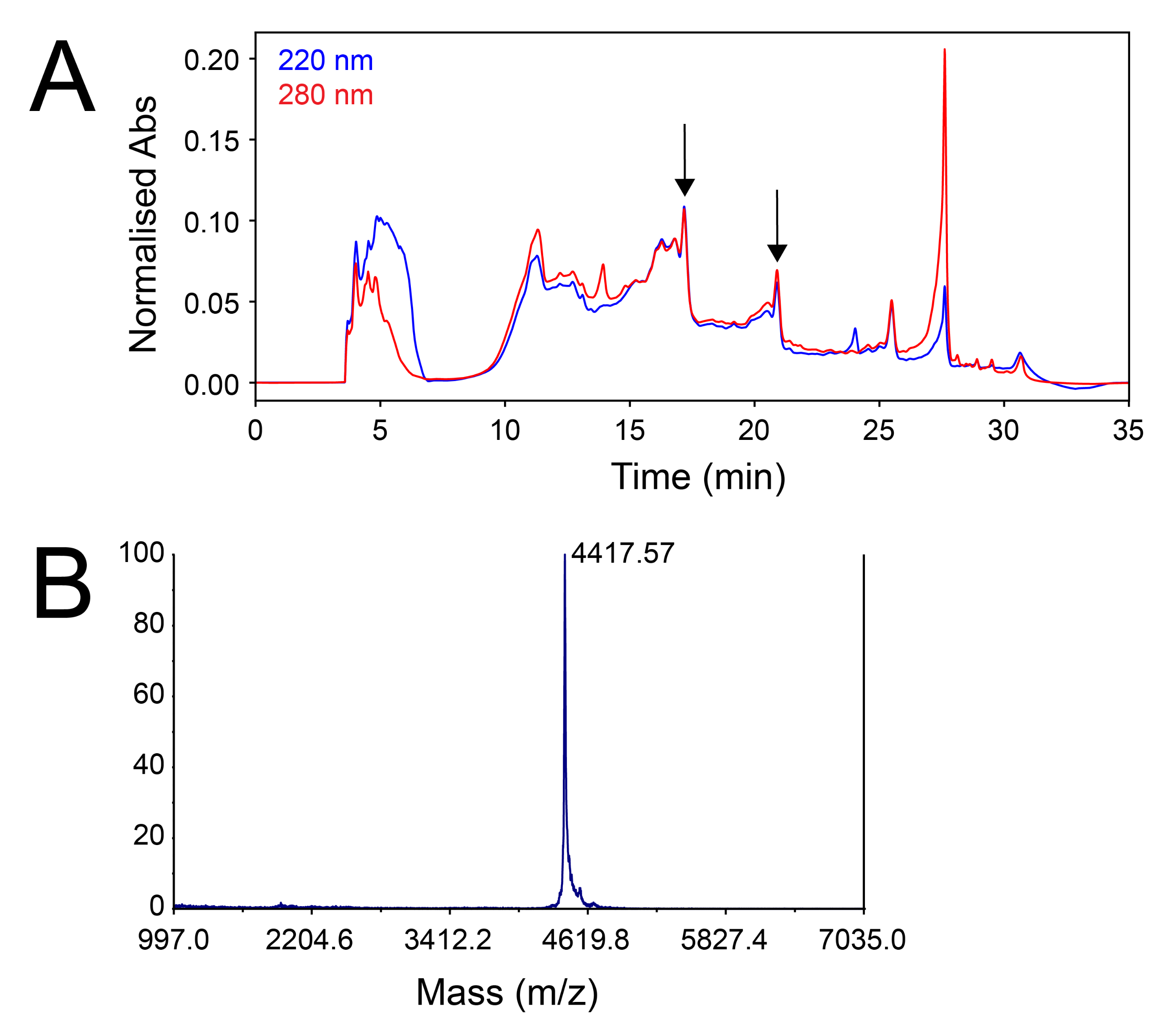
**

**Figure S6. Purification of synthetic peptide 4-44.** (A) Selected HPLC chromatogram of peptide 4-44 showing elution traces monitored at 220 nm (red) and 280 nm (blue). Peaks containing pure peptides are indicated by arrows; (B) selected MALDI-TOF MS spectra of the fraction containing peptide 4-44 with the m/z value indicated.

**
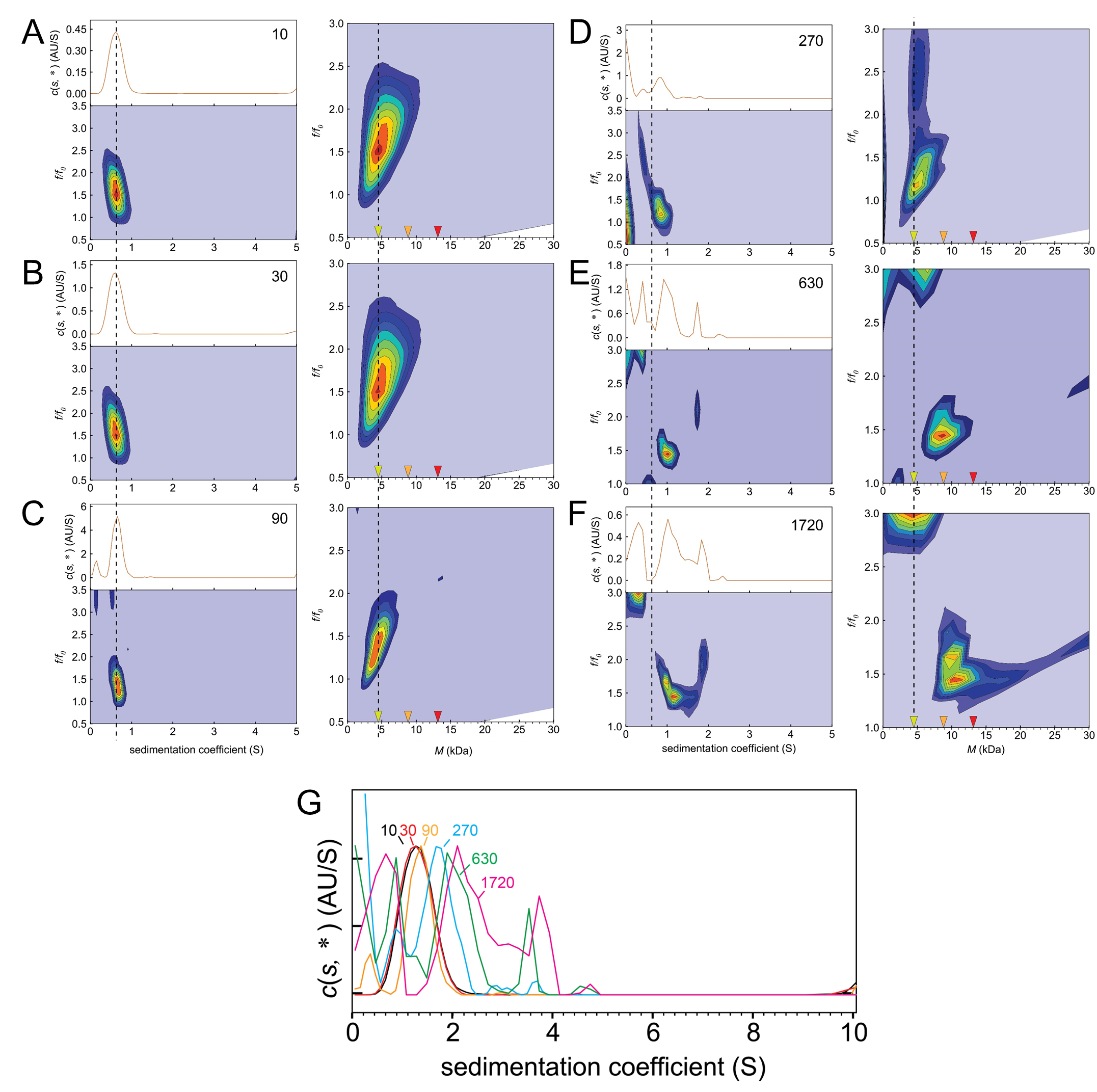
**

**Figure S7. Size-and-shape *c(s, ffo)* distribution of synthetic ZN’ peptide (4-44).** (A-F) Plots of S versus f/fo at the synthetic ZN’ peptide concentrations (µM) shown at the top-right of each panel. The *c(s, *)* projection is shown above each panel; The panels on the right are plots of molecular weight (M) versus f/fo, where the theoretical molecular weights of monomer, dimer, and trimer are indicated with inverted triangles yellow, orange, and red, respectively; (G) the *c(s, *)* projections from panels A-F are overlapped for comparison.

**
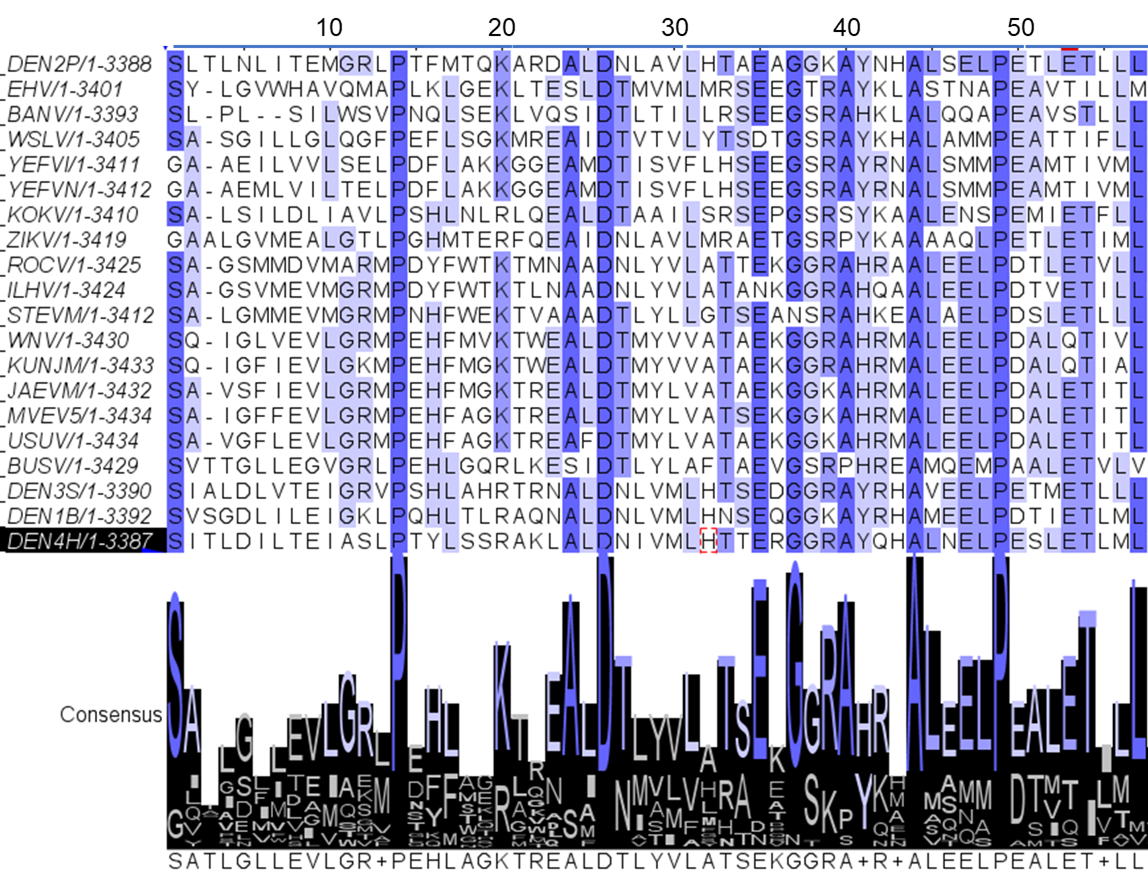
**

**
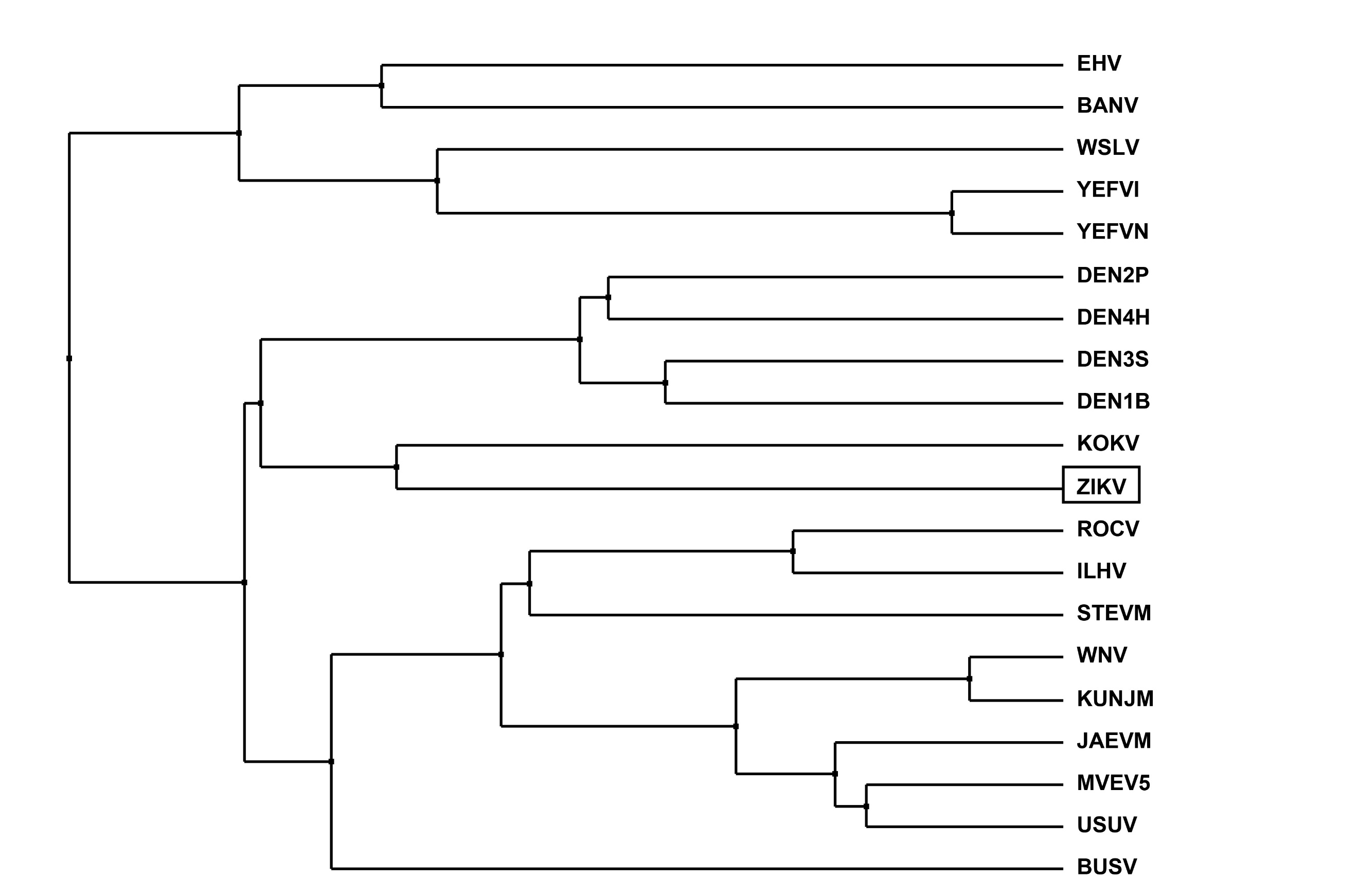
**

**Figure S8.** Jalview [[2](#_ENREF_2)] alignment of NS4A (residues 1-58) in 20 diverse flaviviruses sequences after removing those with >95% redundancy: Zika (ZIKV), Dengue 1-4 (DENV), Yellow Fever (YEFV), Japanese Encephalitis (JAEV), Usutu virus (USUV), Bussuquara virus (BUSV), Rocio virus (ROCV), Banzi (BANV), Edge Hill virus (EHV), Wesselsbron virus (WSLV), Kokobera virus (KOKV), Kunjin virus (KUNV), Ilheus virus (ILHV), West Nile virus (WNV), Saint Louis encephalitis virus (SLEV) and Murray Valley encephalitis virus (MVEV). Columns corresponding to most conserved residues (50% cut-off) are highlighted in blue. Consensus sequence is shown at the bottom, with letter size proportional to conservation. Regions with large clusters of conserved residues are highlighted in red. Lower panel: evolutionary tree of NS4A sequences using BLOSUM62.

1. Waterhouse AM, Procter JB, Martin DMA, Clamp M, Barton GJ. Jalview Version 2—a multiple sequence alignment editor and analysis workbench. Bioinformatics. 2009;25(9):1189-91. doi: 10.1093/bioinformatics/btp033.
